# Assessment of anxiety related to Covid-19 in the elderly in Tunisia

**DOI:** 10.1192/j.eurpsy.2022.782

**Published:** 2022-09-01

**Authors:** M. Moalla, R. Lansari, R. Saida, E. Bergaoui, A. Larnaout, W. Melki

**Affiliations:** Razi Hospital, Psychiatry D, Manouba, Tunisia

**Keywords:** Anxiety, Covid-19, Elderly, Assessment

## Abstract

**Introduction:**

The COVID-19 pandemic has several risks particularly in the elderly, such a high death rate and severe forms.The risk also involves significant psychological distress especially anxiety.

**Objectives:**

Assessment of anxiety symptoms due to COVID 19 in elderly subjects in Tunisia.

**Methods:**

A cross-sectional descriptive study on a sample of 50 people aged between 65 and over. Data was collected using a questionnaire and a COVID Anxiety Rating Scale “Fear of COVID-19 Scale”.

**Results:**

We found that most subjects agreed or strongly agreed that they were afraid of Corona and that 54% agreed or strongly agreed that they felt uncomfortable thinking about the Corona. Physical symptoms like having sweaty hands, or having palpitations related to the fear of having the disease were reported by 24% of the subjects. Almost half of the respondents (46%) were afraid of losing their lives due to Corona. Most subjects (66%) reported that they became nervous or anxious when viewing information posted on social media on Corona. Insomnia related to worries about having COVID was reported in 12% of subjects. We were able to retain that most of the subjects (68%) had a mild level of anxiety and that 30% of the subjects had a moderate level. No subject had severe anxiety.

**Conclusions:**

Low to moderate level of fear due to the COVID 19 pandemic was observed among Tunisian elderly according to this study. This leads us to conclude that assessment of anxiety particularly in seniors should be more systematic.

**Disclosure:**

No significant relationships.

